# Mitochondrial function and oxidative stress in white adipose tissue in a rat model of PCOS: effect of SGLT2 inhibition

**DOI:** 10.1186/s13293-022-00455-x

**Published:** 2022-08-19

**Authors:** Jacob E. Pruett, Steven J. Everman, Ngoc H. Hoang, Faridah Salau, Lucy C. Taylor, Kristin S. Edwards, Jonathan P. Hosler, Alexandra M. Huffman, Damian G. Romero, Licy L. Yanes Cardozo

**Affiliations:** 1 Department of Cell and Molecular Biology, University of Mississippi Medical Center, 2500 N. State Street, Jackson, MS 39216-4505 USA; 2 Department of Medicine, University of Mississippi Medical Center, Jackson, MS USA; 3 Mississippi Center of Excellence in Perinatal Research, University of Mississippi Medical Center, Jackson, MS USA; 4 Women’s Health Research Center, University of Mississippi Medical Center, Jackson, MS USA; 5 Cardio Renal Research Center, University of Mississippi Medical Center, Jackson, MS USA

**Keywords:** Polycystic ovary syndrome, Androgens, Mitochondrial dysfunction, White adipose tissue, Sodium–glucose cotransporter-2

## Abstract

**Background:**

Polycystic ovary syndrome (PCOS), characterized by androgen excess and ovulatory dysfunction, is associated with a high prevalence of obesity and insulin resistance (IR) in women. We demonstrated that sodium–glucose cotransporter-2 inhibitor (SGLT2i) administration decreases fat mass without affecting IR in the PCOS model. In male models of IR, administration of SGLT2i decreases oxidative stress and improves mitochondrial function in white adipose tissue (WAT). Therefore, we hypothesized that SGLT2i reduces adiposity via improvement in mitochondrial function and oxidative stress in WAT in PCOS model.

**Methods:**

Four-week-old female rats were treated with dihydrotestosterone for 90 days (PCOS model), and SGLT2i (empagliflozin) was co-administered during the last 3 weeks. Body composition was measured before and after SGLT2i treatment by EchoMRI. Subcutaneous (SAT) and visceral (VAT) WAT were collected for histological and molecular studies at the end of the study.

**Results:**

PCOS model had an increase in food intake, body weight, body mass index, and fat mass/lean mass ratio compared to the control group. SGLT2i lowered fat mass/lean ratio in PCOS. Glucosuria was observed in both groups, but had a larger magnitude in controls. The net glucose balance was similar in both SGLT2i-treated groups. The PCOS SAT had a higher frequency of small adipocytes and a lower frequency of large adipocytes. In SAT of controls, SGLT2i increased frequencies of small and medium adipocytes while decreasing the frequency of large adipocytes, and this effect was blunted in PCOS. In VAT, PCOS had a lower frequency of small adipocytes while SGLT2i increased the frequency of small adipocytes in PCOS. PCOS model had decreased mitochondrial content in SAT and VAT without impacting oxidative stress in WAT or the circulation. SGLT2i did not modify mitochondrial function or oxidative stress in WAT in both treated groups.

**Conclusions:**

Hyperandrogenemia in PCOS causes expansion of WAT, which is associated with decreases in mitochondrial content and function in SAT and VAT. SGLT2i increases the frequency of small adipocytes in VAT only without affecting mitochondrial dysfunction, oxidative stress, or IR in the PCOS model. SGLT2i decreases adiposity independently of adipose mitochondrial and oxidative stress mechanisms in the PCOS model.

**Supplementary Information:**

The online version contains supplementary material available at 10.1186/s13293-022-00455-x.

## Highlights

1. Androgen excess in PCOS model is associated with decreased markers of mitochondrial content in both subcutaneous and visceral white adipose tissue.
2. Androgen excess in PCOS model is associated with increased frequency of small adipocytes in subcutaneous white adipose tissue while decreasing frequency of small adipocytes in visceral white adipose tissue.
3. SGLT2 inhibition did not modify markers of mitochondrial content or oxidative stress in either subcutaneous or visceral white adipose tissue in PCOS model.
4. SGLT2 inhibition increased frequency of small adipocytes in both subcutaneous and visceral white adipose tissue in control rats; however, SGLT2 inhibition only increased frequency of small adipocytes in visceral white adipose tissue in PCOS model.

**Supplementary Information:**

The online version contains supplementary material available at 10.1186/s13293-022-00455-x.

## Introduction

Polycystic ovary syndrome (PCOS) is the most common endocrine disorder in women of reproductive age, affecting approximately 10% of women in this population [1–3]. Using the Rotterdam Criteria, PCOS is characterized by having two of the following characteristics: androgen excess, ovulatory dysfunction, and a polycystic appearance of the ovaries [4]. PCOS is also highly associated with obesity and obesity-related cardiometabolic complications, such as insulin resistance (IR)/type 2 diabetes mellitus (T2DM), increased blood pressure, hyperleptinemia, and renal injury [4–8]. While 80% of patients with PCOS are overweight or obese [9], there are few effective evidence-based pharmacological agents for treating obesity-associated cardiometabolic complications in PCOS [10–12].

Although is clear that obesity exacerbates the cardiometabolic complications in PCOS women [4], the mechanism by which hyperandrogenemia promotes white adipocytes (WAT) expansion remains unclear. We previously demonstrated that hyperandrogenemia in female rats recapitulates several of the cardiometabolic features observed in women with PCOS, including increased body weight, BMI, fat mass, IR, blood pressure, and albuminuria [13, 14]. The expansion of WAT is present in both the subcutaneous (SAT) and visceral WAT (VAT) [13, 15, 16].

Mitochondria are essential in regulating oxidative stress and energy demands in WAT [17, 18]. Mitochondrial dysfunction occurs when mitochondria fail to provide sufficient ATP for the cell, or when they generate a damaging amount of reactive oxygen species (ROS), or both [19, 20]. Women with PCOS have decreased mitochondrial content or volume in circulating leukocytes [21, 22]. Furthermore, in a PCOS mouse model, oocytes have decreased inner mitochondrial membrane potential, altered mitochondrial structure, and increased ROS [23]. Whether mitochondrial dysfunction in WAT underlies the pathophysiology of obesity-associated cardiometabolic complications in PCOS remains unknown.

As reviewed by Harper et al. [24], mitochondria are a major source of cellular ROS. Much of the superoxide, a main ROS, is converted to hydrogen peroxide by cytosolic (SOD1) and mitochondrial (SOD2) superoxide dismutase. Superoxide and hydrogen peroxide interact to produce highly reactive hydroxyl radical that modifies lipids, DNA, and protein. Hydroxyl radical causes lipid peroxidation, which forms isoprostanes [25]. Lipid peroxidation can lead to inflammation, tissue dysfunction, and an unhealthy expansion of WAT [25]. A recent report showed that testosterone administration decreased SOD and catalase activities in retroperitoneal WAT in male rats [26]. Even when matched for body mass index, women with PCOS also have decreased circulating antioxidant capacity and increased markers of oxidative stress [27]. Whether excess androgens in PCOS cause oxidative stress and mitochondrial dysfunction in WAT in PCOS is unknown.

Sodium–glucose cotransporter-2 (SGLT2) reabsorbs glucose in the proximal tubule of the nephron, being responsible for about 90% of glucose reabsorption in the nephron [28, 29]. The clinical indications for SGLT2 inhibitors have been expanding to a variety of diseases such as type 2 diabetes mellitus, heart failure, and chronic kidney disease [30–32]. A recent small clinical trial demonstrated that administration of the SGLT2 inhibitor empagliflozin (EMPA) decreased body weight (BW), body mass index (BMI), and fat mass compared to metformin in PCOS women [11]. Interestingly, these benefits occurred without modifying plasma dihydrotestosterone or fasting plasma glucose, insulin, or cholesterol. Furthermore, there is no known SGLT2 expression in white adipose tissue that could explain a direct effect of SGLT2 inhibition on adipose tissue [33]. Using a model of PCOS, the hyperandrogenemic female rat, we recently showed that the SGLT2 inhibitor EMPA decreased fat mass and leptin levels [14]. Similar to PCOS women [11], we demonstrated that this decrease in fat mass occurred without any decreases in fasting glucose, insulin, cholesterol, or triglycerides, nor did we observe decreases in lean mass, food intake, hemoglobin A1c, or ketonuria [14]. The mechanisms by which SGLT2 inhibitors (SGLT2i) decrease the fat mass in PCOS remain unclear.

A recent study by Wei et al. found that in male mice with T2DM, SGLT2i decreased fat mass in associated with improvement in mitochondrial function [34]. Furthermore, they reported that SGLT2 inhibition in adipocytes was associated with increased expression of peroxisome proliferator-activated receptor-γ coactivator 1-α (PGC1α) and nuclear respiratory factor 1 (NRF1) expression. PGC1α enhances the action of peroxisome proliferator-activated receptor-γ (PPARγ) to act as a key regulator of adipogenesis and mitochondrial biogenesis [35]. NRF1 is under the control of PGC1α and regulates both mitochondrial biogenesis [35] and increases oxidative phosphorylation via changes in the complex IV [36]. Furthermore, Wei et al. also found that SGLT2i treatment led to increased expression of rate-limiting enzymes of mitochondrial fatty acid oxidation, including carnitine palmitoyltransferase 1b (CPT1B) and medium-chain acyl-CoA dehydrogenase (MCAD) [34]. The rate-limiting step of long-chain fatty acid oxidation is CPT1A and CPT1B transport of fatty acids into the mitochondria to begin fatty acid oxidation [37]. Whether SGLT2i improves mitochondrial function and oxidative stress in WAT in PCOS is unknown.

In this study, we hypothesized that androgens cause expansion and dysfunction of WAT in SAT and VAT via mitochondrial dysfunction and oxidative stress and that SGLT2 inhibition reverses these processes in the PCOS model.

## Materials and methods

### Experimental model of PCOS and empagliflozin administration

Three-week-old female Sprague Dawley rats were obtained from Envigo (Indianapolis, IN, USA). At 4 weeks of age, rats were randomly assigned to be implanted subcutaneously with continuous-release of dihydrotestosterone (DHT) pellets (7.5 mg/90 days; Innovative Research of America, Sarasota, FL, USA) or sham surgery (Control) as we previously reported [14]. Rats were maintained on a standard rat chow diet (Teklad 22/5 Rodent Diet #8640; Envigo, Indianapolis, IN, USA), housed in temperature-controlled rooms with ad libitum food and water, and a constant light/dark cycle (12 h/12 h). Animals were followed for 90 days. All experimental protocols were performed following the National Institutes of Health Guide for the Care and Use of Laboratory Animals, 8th Edition, 2011, and approved by the Institutional Animal Care and Use Committee of the University of Mississippi Medical Center.

As we previously reported [14], the sodium–glucose cotransporter-2 inhibitor, EMPA (10 mg/kg/day, AChemBlock, CA, USA), was administered in drinking water at a dose shown to be effective in lowering BP and hemoglobin A1c in other rodent models [38, 39]. EMPA treatment was administrated during the last 3 weeks of the experimental protocol. During EMPA treatment, fluid intake was measured daily, and body weight was measured twice a week to dose EMPA appropriately each day.

### Anthropometric measurements, metabolic assays, and oxidative stress assays

At 12 weeks of age (1 week before EMPA treatment) and at 16 weeks of age (after 3 weeks of EMPA treatment), body composition (fat and lean mass) was measured in duplicate before and after treatment by EchoMRI (4in1-900 Body Composition Analyzer, EchoMRI, Houston, TX, USA), as we previously reported [14]. At 12 weeks of age and 16 weeks of age, rats were also placed individually into metabolic cages for 24-h urine collection. Urine was centrifuged at 2100×*g* for 20 min at 4 °C, aliquoted, and centrifuged again at 2100×*g* for 20 min at 4 °C. Supernatants were stored at − 80 °C. Urine glucose and creatinine levels were measured using VET Axcel Chemistry Analyzer as previously reported [14]. Total urinary 15-isoprostane F_2t_ excretion before and after EMPA treatment was assessed by ELISA after β-glucuronidase treatment per the manufacturer’s instructions (EA85, Oxford Biomedical Research, Oxford, MI, USA). During EMPA treatment, food intake was measured daily. Net glucose balance was calculated by subtracting 24-h intake of available carbohydrates in the food and 24-h urinary glucose excretion, and it is reported as mg/day. Isoprostane excretion was normalized by creatinine and reported as a urinary isoprostane to creatinine ratio (UICR) in ng/mg. At the end of experimental period, body length (nose–anus length) was measured to calculate body mass index (BMI). Arterial heparinized plasma was collected at euthanasia for total antioxidant capacity according to the manufacturer’s instructions (TA02, Oxford Biomedical Research, Oxford, MI, USA) and reported as copper reducing equivalents (CRE) μM.

### Tissue collection

At 16 weeks of age (3 weeks after EMPA treatment), rats were euthanized under isoflurane anesthesia for white adipose tissue (WAT) collection. Two visceral WAT (retroperitoneal and mesenteric) depots were collected. Subcutaneous (inguinal) WAT was also obtained as subcutaneous WAT is known to be metabolically distinct from visceral WAT (VAT) [40]. A portion of subcutaneous WAT (SAT), retroperitoneal WAT (rWAT), and mesenteric WAT (mWAT) were snap-frozen in liquid nitrogen for mRNA and protein expression. For histology, WAT depots were fixed in 10% formalin for 24 h followed by washing in 70% ethanol and embedment in paraffin. Another portion of SAT and rWAT were snap-frozen in liquid nitrogen for mitochondrial activity assays and the 2-thiobarbituric acid reactive substances (TBARS) assay.

### mRNA expression quantification

In the three WAT depots, adipose total RNA was extracted with TRI-Reagent (Molecular Research Center, Inc., Cincinnati, OH, USA), DNAse-treated with Turbo DNA-free kit (ThermoFisher Scientific, Waltham, MA, USA), and quantified. One microgram of RNA was reverse transcribed with SuperScript IV reverse transcriptase (ThermoFisher Scientific, Waltham, MA, USA) as we previously reported [14]. Gene expression was quantified by quantitative RT-PCR using TaqMan technology and Luna Universal Probe qPCR Master Mix (New England Biolabs, Ipswich, MA, USA). TaqMan Assays (ThermoFisher Scientific, Waltham, MA, USA) are reported in Table 1. PCR product quantification was performed by the ΔΔCt relative quantification method and expressed as log2 arbitrary units (AU) normalized against the geometric mean of three housekeeping genes (β-actin, β-2-microglobulin, and glyceraldehyde 3-phosphate dehydrogenase) and standardized to untreated control rats.

**Table 1:** TaqMan assay IDs

Gene name	Gene symbol	TaqMan assay ID
Carnitine palmitoyltransferase 1A (CPT1A)	*Cpt1a*	Rn00580702_m1
Carnitine palmitoyltransferase 1B (CPT1B)	*Cpt1b*	Rn00682395_m1
Medium-chain acyl-CoA dehydrogenase (MCAD)	*Acadm*	Rn00566390_m1
Cytosolic superoxide dismutase (SOD1)	*Sod1*	Rn00566938_m1
Mitochondrial superoxide dismutase (SOD2)	*Sod2*	Rn00690588_g1
Catalase	*Cat*	Rn00560930_m1
Peroxisome proliferator-activated receptor-γ (PPARγ)	*Pparg*	Rn00440945_m1
PPARγ coactivator 1-α (PGC1α)	*Ppargc1a*	Rn00580241_m1
Nuclear respiratory factor 1 (NRF1)	*Nrf1*	Rn01455958_m1
Sodium–glucose cotransporter-2 (SGLT2)	*Slc5a2*	Rn00574917_m1
β-Actin	*Actb*	Rn00667869_m1
β-2-microglobulin	*B2m*	Rn00560865_m1
Glyceraldehyde 3-phosphate dehydrogenase	*Gapdh*	Rn01775763_g1

### Protein expression quantification

Western blotting was performed as previously reported [14]. WAT samples were homogenized in radioimmunoprecipitation assay buffer supplemented with Halt protease and phosphatase inhibitor cocktail (ThermoFisher Scientific, Waltham, MA, USA). Total protein was quantified with bicinchoninic acid protein assay kit (ThermoFisher Scientific, Waltham, MA, USA). Fifty micrograms of total protein were separated by SDS-PAGE with 12% Criterion TGX Stain-Free Precast Gels (Bio-Rad, Hercules, CA, USA) and transferred to LF-PVDF membranes (Millipore, Burlington, MA, USA). Blotted membranes were processed and imaged for stain-free technology quantification. Membranes were blocked with 5% nonfat dry milk in Tris-buffered saline containing 0.1% Tween 20 (TBST) for 1 h at room temperature. Membranes were then incubated in anti-SOD1 (1:30,000 in SAT and 1:3000 in rWAT and mWAT; E4G1H; Cell Signaling Technology, Inc., Danvers, MA, USA), anti-SOD2 (1:150,000 in SAT and 1:30,000 in rWAT and mWAT; D3X8F, Cell Signaling Technology, Inc., Danvers, MA, USA), or anti-catalase (1:50,000 in SAT and 1:10,000 in rWAT and mWAT; D5N7V, Cell Signaling Technology, Inc., Danvers, MA, USA) primary antibodies overnight at 4 °C. Then, membranes were probed with horseradish peroxidase-conjugated goat anti-rabbit secondary antibody (1:20,000; Jackson ImmunoResearch, West Grove, PA, USA, 111-035-003) for 1 h at room temperature. Detection by chemiluminescence was performed with SuperSignal West Pico PLUS (ThermoFisher Scientific, Waltham, MA, USA). Digital images were acquired with the ChemiDoc MP image system (Bio-Rad, Hercules, CA, USA) and quantified with Image Lab 6 (Bio-Rad, Hercules, CA, USA). Protein expression was normalized to total protein content detected by stain-free technology as we previously reported [14].

### Adipocyte cross-sectional area determination

WAT depot samples were cut into 4-µm sections and then stained with hematoxylin and eosin. Images were acquired at 40× magnification using an Olympus BX63 microscope with cellSens Dimensions software (Olympus, Tokyo, Japan). The adipocyte cross-sectional area of at least 100 cells per rat and WAT depot were quantified using the Adiposoft plugin for ImageJ by investigators blinded to the sample identity [41, 42]. Blinded investigators went through each image to ensure proper identification of adipocytes and removal of false positives by Adiposoft. GraphPad Prism (GraphPad Software Inc., La Jolla, CA, USA) was used to calculate the relative frequency of adipocyte area (bins of 400 μm^2^ for SAT, of 150 μm^2^ for rWAT, and 200 μm^2^ for mWAT). Adipocytes less than 600 μm^2^ were considered small, adipocytes between 600 and 1200 μm^2^ were considered medium, and adipocytes above 1200 μm^2^ were considered large.

### Mitochondrial complexes activities and TBARS assay

Upon euthanasia, subcutaneous and visceral (retroperitoneal) white adipose tissues were snap-frozen in liquid nitrogen and stored at − 80 °C. The tissue was sectioned on dry ice, then minced with a razor blade. The tissue was then homogenized on ice with a Dounce homogenizer and a Teflon pestle at a ratio of ~ 100 mg tissue per 750 μL of extraction buffer (50 mM Tris–HCl, 100 mM KCl, 0.5 mM EDTA, 0.2% dodecylmaltoside, pH 7.4). Homogenates were spun at 17,000×*g* at 4 °C for 5 min, and the supernatant was taken for citrate synthase and complex IV activities and TBARS assay. Citrate synthase activity, a marker of mitochondrial content [43], was measured by spectrophotometer in individual cuvettes in duplicate similar to as previously described [44, 45]. The reaction condition was 1 mM DNTB and 0.3 mM acetyl CoA with sample (50 μL of sample for SAT and 30 μL of sample for VAT) and water added up to 200 μL final volume at 37 °C. The reaction was initiated with 0.5 mM oxaloacetate. No additional detergent was added to permeabilize the mitochondria due to the presence of dodecylmaltoside in the sample. Complex IV activity, a marker of oxidative phosphorylation capacity [43], was measured in duplicate by respirometry (Oroboros Oxygraph-2k) similar to as previously described [46]. The reaction condition was 50 mM Tris, 8 mM KCl, 1 mM EDTA, 0.3 mM TMPD, 25 μM cytochrome *c*, and 16 μg/μL catalase at 37 °C. The reaction was initiated by the addition of 90 μL of the sample, and the reaction was stopped with 50 mM ZnSO_4_ and 50 mM MgCl_2_. The TBARS assay was done according to the manufacturer’s instructions to measure total malondialdehyde (FR40, Oxford Biomedical Research, Oxford, MI, USA). Samples were normalized to protein content as determined by the DC Protein assay (Bio-Rad, Hercules, CA, USA). Citrate synthase activity, complex IV activity, and TBARS assay data are reported as nmol/min/mg, nmol e^−^/min/mg, and nmol/mg, respectively.

### Statistical analyses

All data are expressed as mean ± SEM. Data were analyzed by two-way analysis of variance (ANOVA) or by two-way repeated-measures ANOVA followed by post hoc multiple comparisons using Tukey’s test. Isoprostane excretion was analyzed by three-way ANOVA corrected post hoc for multiple comparisons using Tukey’s test to compare between 12 weeks of age and 16 weeks of age. Differences were considered statistically significant at *P* < 0.05. Statistical analyses were performed with GraphPad Prism 9 software package version 9.0.0 (GraphPad Software Inc., La Jolla, CA, USA).

## Results

### Anthropomorphic measures and glucose homeostasis in PCOS model: effect of EMPA

**Fig. 1:**
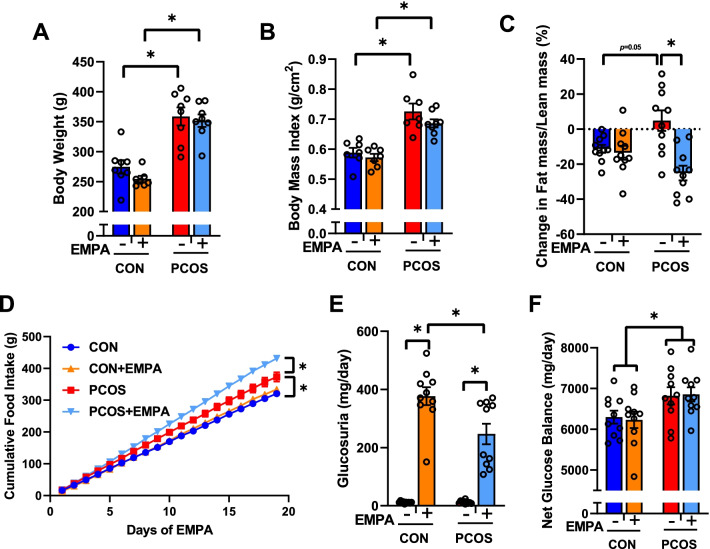
Effect of empagliflozin (EMPA) on anthropomorphic and glucose measures in PCOS. Effect of EMPA on **A** body weight, **B** body mass index, **C** change in fat mass/lean mass between 12 and 16 weeks of age, **D** cumulative food intake, **E** glucosuria, and **F** net glucose balance at 3 weeks of EMPA. Data are expressed as mean ± SEM. Data were analyzed by two-way ANOVA with (**D**) or without (**A**–**C**, **E**, **F**) repeated measures, followed by Tukey post hoc tests. Significant interaction was observed for change in fat mass/lean mass, cumulative food intake, and glucosuria. *P < 0.05. *n* = 6–10 per group

As seen in Fig. 1A–D, PCOS rats had increased body weight (358.8 ± 15.0 vs 274.6 ± 11.2 g, *P* < 0.0001), increased BMI (0.726 ± 0.026 vs 0.588 ± 0.016 g/cm^2^, *P* < 0.0001), increased fat mass/lean mass (4.82 ± 5.85 vs − 11.28 ± 2.27%, *P* = 0.05), and food intake compared to controls (373.0 ± 14.7 vs 320.8 ± 5.2 g, *P* < 0.01). By the end of the experimental period, fat mass/lean mass was decreased in PCOS + EMPA rats compared to untreated PCOS rats (− 25.08 ± 4.17 vs 4.82 ± 5.85%, *P* < 0.0001, Fig. 1C) from 12 to 16 weeks of age, and EMPA further augmented food intake significantly only in PCOS (431.3 ± 8.0 vs 373.0 ± 14.7 g, *P* < 0.01, Fig. 1D). As shown in Fig. 1E, both EMPA-treated groups had significantly increased urinary glucose excretion, with CON + EMPA rats excreting more glucose than PCOS + EMPA rats (377 ± 31 vs 247 ± 35 mg/day, *P* < 0.01). The net glucose balance was unchanged in both EMPA-treated groups, suggesting that they were equally able to compensate for their glucosuria to match their respective untreated cohort, as seen in Fig. 1F.

### Adipocyte cross-sectional area in PCOS model: effect of EMPA

**Fig. 2:**
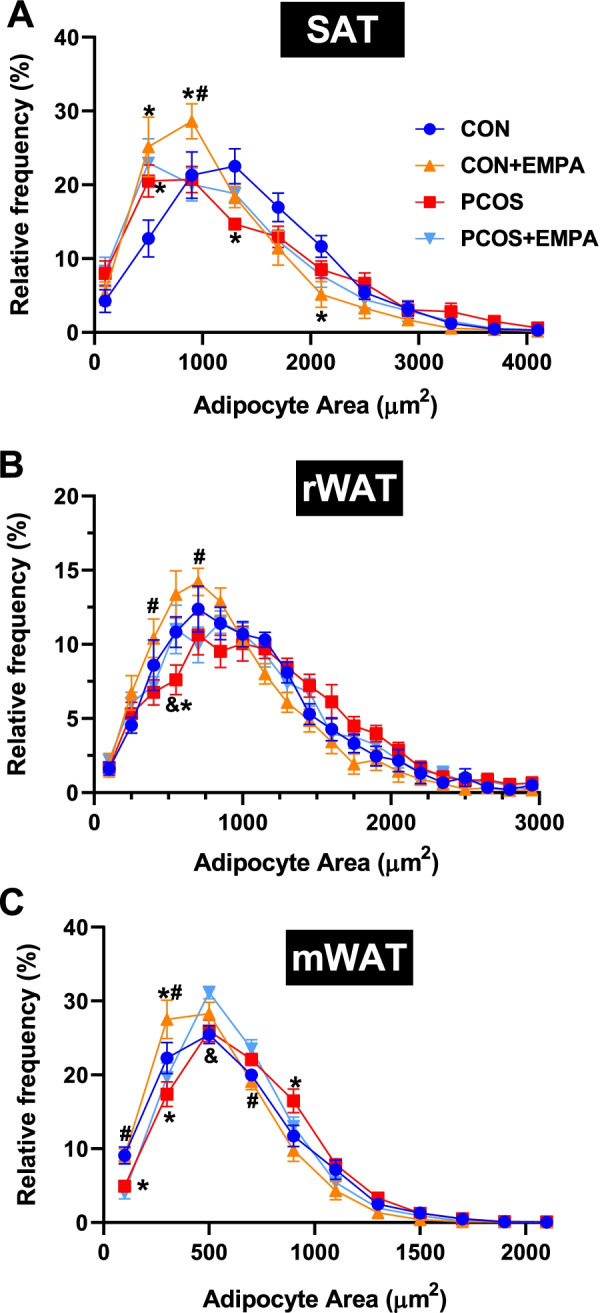
Effect of EMPA on adipocyte cross-sectional area in white adipose depots in PCOS. Effect of EMPA on the relative frequency on adipocyte areas and average adipocyte area in **A** subcutaneous white adipose tissue, **B** visceral (retroperitoneal), and **C** visceral (mesenteric) white adipose tissue. Subcutaneous, retroperitoneal, and mesenteric adipocytes were binned every 400, 150, and 200 microns, respectively. Data were analyzed by two-way ANOVA followed by Tukey post hoc tests. Significant interactions were observed in all three depots with adipocyte area frequency, but there were no significant interactions with average adipocyte cross-sectional area in any of the three depots. *P < 0.05 compared to controls, ^#^*P* < 0.05 CON + EMPA compared to PCOS + EMPA, ^&^*P* < 0.05 PCOS compared to PCOS + EMPA. *n* = 8–10 per group

The effect of EMPA and DHT on adipocyte cross-sectional area varied based on whether the WAT depot was subcutaneous or visceral. In the SAT (Fig. 2A), compared to controls, PCOS had a higher frequency of adipocytes at 500 μm^2^ (20.5 ± 2.2 vs 12.7 ± 2.5%, *P* < 0.01) with lower frequency at 1300 μm^2^ (14.7 ± 0.8 vs 22.5 ± 2.4%, *P* < 0.01). In the control group, EMPA increased frequencies of adipocytes at 500 μm^2^ (25.1 ± 4.0 vs 12.7 ± 2.5%, *P* < 0.0001) and 900 μm^2^ (28.6 ± 2.4 vs 21.3 ± 3.1%, *P* < 0.05) while decreasing frequency at 2100 μm^2^ (5.2 ± 1.7 vs 11.7 ± 1.5%, *P* < 0.05). The effect of EMPA on SAT was blunted in PCOS model.

In the rWAT (Fig. 2B), PCOS had a lower frequency of small adipocytes around 550 μm^2^ compared to controls (7.6 ± 1.0 vs 10.8 ± 1.0%, *P* < 0.05). EMPA in PCOS increased the frequency of small adipocytes around 550 μm^2^ compared to untreated PCOS (11.0 ± 1.6 vs 7.6 ± 1.0%, *P* < 0.01) without affecting the control group.

In mWAT (Fig. 2C), compared to controls, PCOS had a lower frequency of small adipocytes around 100 μm^2^ (4.9 ± 0.5 vs 9.1 ± 1.2%, *P* < 0.05) and 300 μm^2^ (17.4 ± 1.7 vs 22.3 ± 2.1%, *P* < 0.01), and a higher frequency of medium adipocytes around 900 μm^2^ (16.5 ± 1.6 vs 11.7 ± 1.4%, *P* < 0.01). In PCOS, EMPA increased relative frequency of small adipocytes at 500 μm^2^ (31.1 ± 0.8 vs 26.0 ± 1.5%, *P* < 0.01). In controls, EMPA increased relatively frequency of small adipocytes at 300 μm^2^ (27.5 ± 2.6 vs 22.3 ± 2.1%, *P* < 0.01). Representative images of SAT, rWAT, and mWAT adipocytes can be found in Additional file 1: Figure S1A–C.

### Antioxidant enzyme gene and protein expression in PCOS model: effect of EMPA

**Fig. 3:**
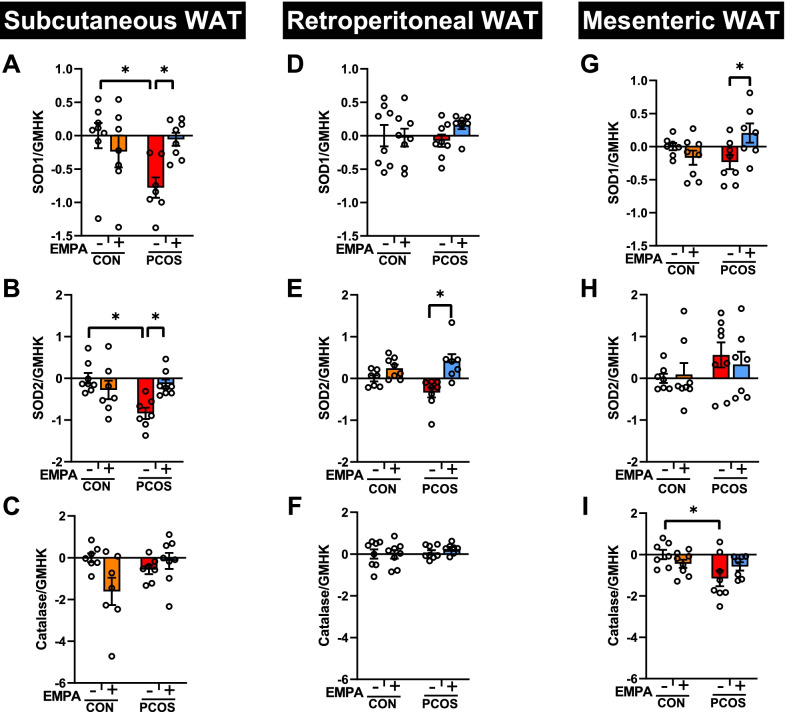
Effect of EMPA on mRNA expression on antioxidant enzymes in white adipose depots in PCOS. Effect of EMPA on subcutaneous white adipose tissue (WAT) mRNA expression of **A** cytosolic superoxide dismutase (SOD1), **B** mitochondrial superoxide dismutase (SOD2), and **C** catalase; retroperitoneal WAT mRNA expression of **D** SOD1, **E** SOD2, and **F** catalase; and mesenteric WAT mRNA expression of **G** SOD1, **H** SOD2, and **I** catalase after 3 weeks of EMPA treatment. Expression was normalized by the geometric mean of three housekeeping genes (GMHK) and standardized to untreated control rats. Log2 values are expressed as mean ± SEM. Data were analyzed by two-way ANOVA followed by Tukey post hoc tests. Significant interactions were observed for SOD1, SOD2, and catalase in subcutaneous WAT, in SOD2 in retroperitoneal WAT, and in SOD1 in mesenteric WAT. *P < 0.05. *n* = 7–8 per group

SGLT2 mRNA was not expressed in SAT, rWAT, or mWAT in any of the four experimental groups (data not shown). In SAT (Fig. 3A–C), compared to controls, PCOS rats had decreased SOD1 (− 0.78 ± 0.15 vs 0.00 ± 0.19, *P* < 0.05) and SOD2 (− 0.84 ± 0.14 vs 0.00 ± 0.13, *P* < 0.01) mRNA expression, with no significant effect on catalase mRNA expression. EMPA in PCOS increased SOD1 (− 0.06 ± 0.10 vs − 0.78 ± 0.15, *P* < 0.05) and SOD2 (− 0.14 ± 0.11 vs − 0.84 ± 0.14, *P* < 0.05) mRNA expression, with no impact on catalase. EMPA had no significant effect SOD1, SOD2, or catalase mRNA expression in SAT in controls. In rWAT (Fig. 3D–F), DHT administration had no significant effect on SOD1, SOD2, or catalase mRNA expression. However, EMPA increased SOD2 expression in PCOS (0.41 ± 0.17 vs − 0.34 ± 0.12, *P* < 0.001) in rWAT. PCOS had decreased catalase mRNA expression compared to controls (− 1.15 ± 0.38 vs 0.00 ± 0.22, *P* < 0.05), though SOD1 and SOD2 were unchanged in mWAT in PCOS model (Fig. 3G–I). EMPA increase SOD1 mRNA expression in PCOS in the mWAT (0.20 ± 0.15 vs − 0.23 ± 0.11, *P* < 0.05), though EMPA had no effect on SOD2 or catalase mRNA in this depot.

**Fig. 4:**
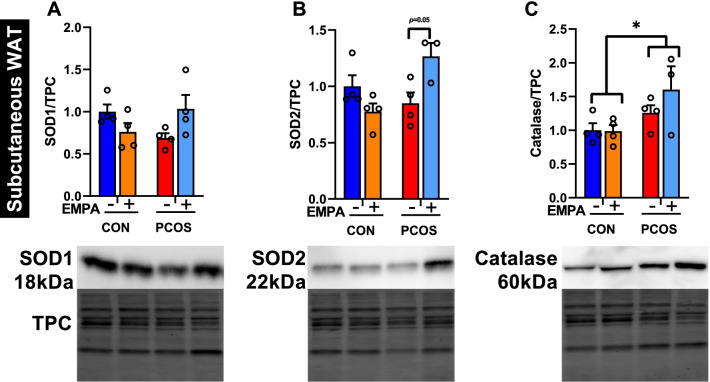
Effect of EMPA on protein expression on antioxidant enzymes in subcutaneous white adipose tissue in PCOS. Effect of EMPA on subcutaneous white adipose tissue (WAT) protein expression of **A** cytosolic superoxide dismutase (SOD1, ~ 18 kDa), **B** mitochondrial superoxide dismutase (SOD2, ~ 22 kDa), and **C** catalase (~ 60 kDa) after 3 weeks of EMPA treatment. Data were normalized by total protein content (TPC). Data are expressed as mean ± SEM and were analyzed by two-way ANOVA followed by Tukey post hoc tests. Significant interactions were observed for SOD1 and SOD2 in subcutaneous WAT. *P < 0.05. *n* = 3–4 per group

In SAT (Fig. 4A), SOD1 protein expression trended to decrease in PCOS, though there were no significant differences in the post hoc analysis. There was a significant interaction in SOD1 protein expression (*P* < 0.05) between EMPA and DHT, as there was in the gene expression, with EMPA seemingly increasing SOD1 protein expression in PCOS while decreasing in controls. Similar to SOD1, SOD2 protein expression matched the trends of its gene expression, and the interaction was significant in both the gene (*P* < 0.01) and protein (*P* < 0.01) expression. EMPA increased SOD2 protein expression (1.27 ± 0.12 vs 0.85 ± 0.10, *P* = 0.056) in PCOS while decreasing SOD2 expression in controls. Meanwhile, catalase protein (Fig. 4C) was increased by DHT overall (*P* < 0.05) with no significant impact by EMPA in either group.

In rWAT, neither DHT nor EMPA had a significant impact on SOD1, SOD2, or catalase protein expression (Additional file 1: Figure S2). In mWAT, similar to the SAT, catalase protein expression was increased overall by DHT (*P* < 0.05) (Fig. 3). However, neither DHT nor EMPA had a significant impact on SOD1 or SOD2 protein expression in mWAT.

### Systemic and WAT oxidative stress in PCOS model: effect of EMPA

**Fig. 5:**
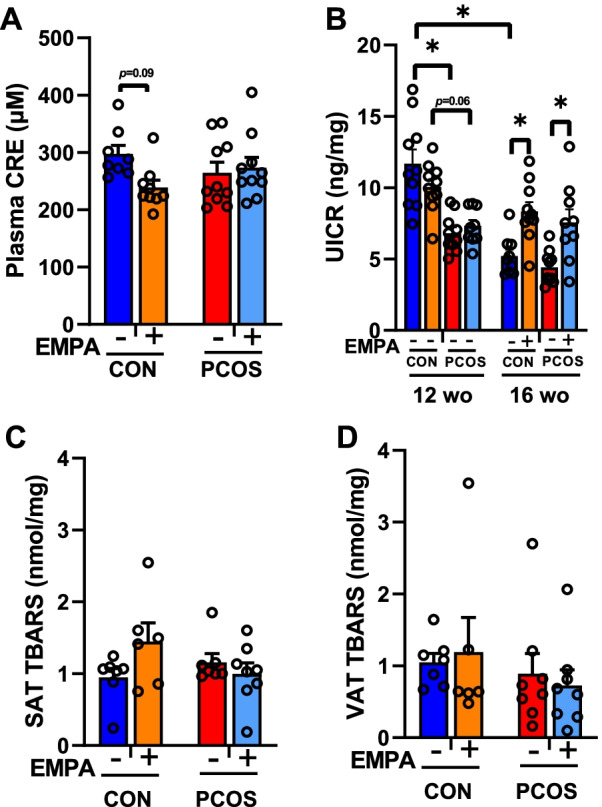
Effect of EMPA on systemic and white adipose tissue (WAT) oxidative stress markers in PCOS. Effect of EMPA on **A** heparinized plasma total antioxidant capacity expressed as copper reducing equivalents (CRE) after 3 weeks of EMPA, and **B** urinary isoprostane to creatinine ratio (UICR) before EMPA treatment (12 weeks of age or 12 wo) and after 3 weeks of EMPA treatment (16 weeks of age or 16 wo). Effect of EMPA on **C** subcutaneous WAT (SAT) 2-thiobarbituric acid reactive substances (TBARS) and **D** visceral (retroperitoneal) WAT (VAT) TBARS after 3 weeks of EMPA treatment. TBARS data are normalized to protein content. Data are expressed as mean ± SEM and were analyzed by two-way ANOVA (**A**, **C**, **D**) or by three-way ANOVA (**B**) followed by Tukey post hoc tests. A significant interaction was only observed for plasma total antioxidant capacity. *P < 0.05. *n* = 6–10 per group

With plasma total antioxidant capacity, there was a significant interaction between DHT and EMPA (*P* < 0.05), though there were no significant post hoc comparisons (Fig. 5A). While EMPA slightly lowered total antioxidant capacity in controls (239 ± 12 vs 297 ± 15 CRE μM, *P* = 0.09), EMPA did not decrease total antioxidant capacity in PCOS (273 ± 18 vs 264 ± 18 CRE μM, *P* = 0.98). At 12 weeks of age (Fig. 5B), before EMPA treatment, we confirmed that both groups of control rats had equal urinary isoprostanes to creatine ratio (UICR) at baseline, which was also similar in both groups of PCOS rats. We also found that 12-week-old control rats had increased UICR compared to 12-week-old PCOS rats (11.7 ± 1.0 vs 6.8 ± 0.4 ng/mg, *P* < 0.0001). By 16 weeks of age, untreated controls had decreased their UICR by about half (5.2 ± 0.5 vs 11.7 ± 1.0 ng/mg, *P* < 0.0001). Untreated PCOS rats also reduced their UICR from 12 to 16 weeks of age, though this was not statistically significant. However, neither control nor PCOS rats treated with EMPA did not have such a reduction in UICR in this time period. In SAT and VAT, neither DHT nor EMPA had a significant impact on TBARS (Fig. 5C, D).

### Gene expression of regulators of mitochondrial biogenesis in PCOS model: effect of EMPA

**Fig. 6:**
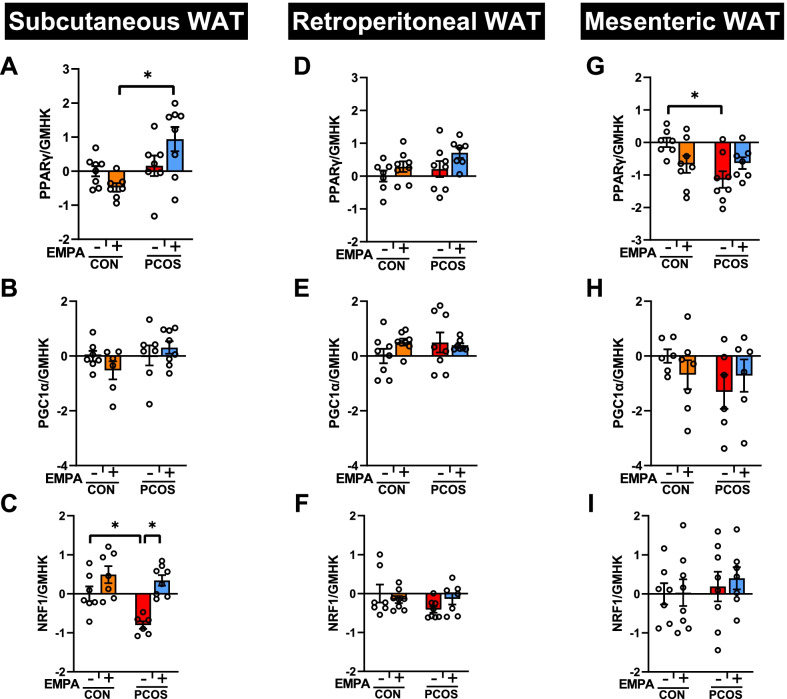
Effect of EMPA on mRNA expression on regulators of mitochondrial biogenesis in white adipose depots in PCOS. Effect of EMPA on subcutaneous white adipose tissue (WAT) mRNA expression of **A** Peroxisome proliferator-activated receptor-γ (PPARγ), **B** PPARγ coactivator 1-α (PGC1α), and **C** Nuclear respiratory factor 1 (NRF1); retroperitoneal WAT mRNA expression of **D** PPARγ, **E** PGC1α, and **F** NRF1; and mesenteric WAT mRNA expression of **G** PPARγ, **H** PGC1α, and **I** NRF1 after 3 weeks of EMPA treatment. Expression was normalized by the geometric mean of three housekeeping genes (GMHK) and standardized to untreated control rats. Log2 values are expressed as mean ± SEM. Data were analyzed by two-way ANOVA followed by Tukey post hoc tests. Significant interactions were observed for PPARγ in both subcutaneous and mesenteric WATs. *n* = 6–8 per group

In SAT (Fig. 6A–C), PCOS rats NRF1 gene expression was downregulated compared to controls (− 0.80 ± 0.10 vs 0.00 ± 0.19, *P* < 0.05), with no changes in PPARγ or PGC1α expression. EMPA in PCOS upregulated NRF1 (0.34 ± 0.14 vs − 0.80 ± 0.10, *P* < 0.001). PCOS + EMPA rats also had increased PPARγ mRNA expression compared to CON + EMPA (0.94 ± 0.36 vs − 0.48 ± 0.13, *P* < 0.01). In SAT, EMPA had no other effect on these three genes. In contrast, in rWAT (Fig. 6D–F), neither DHT nor EMPA had a significant effect on PPARγ, PGC1α, or NRF1. In mWAT (Fig. 6G–I), PCOS significantly reduced PPARγ mRNA expression compared to controls (− 1.14 ± 0.26 vs 0.00 ± 0.15, *P* < 0.01). However, PCOS and EMPA had no other effect on PPARγ, PGC1α, or NRF1 in mWAT, similar to rWAT.

### Mitochondrial function in PCOS model: effect of EMPA

**Fig. 7:**
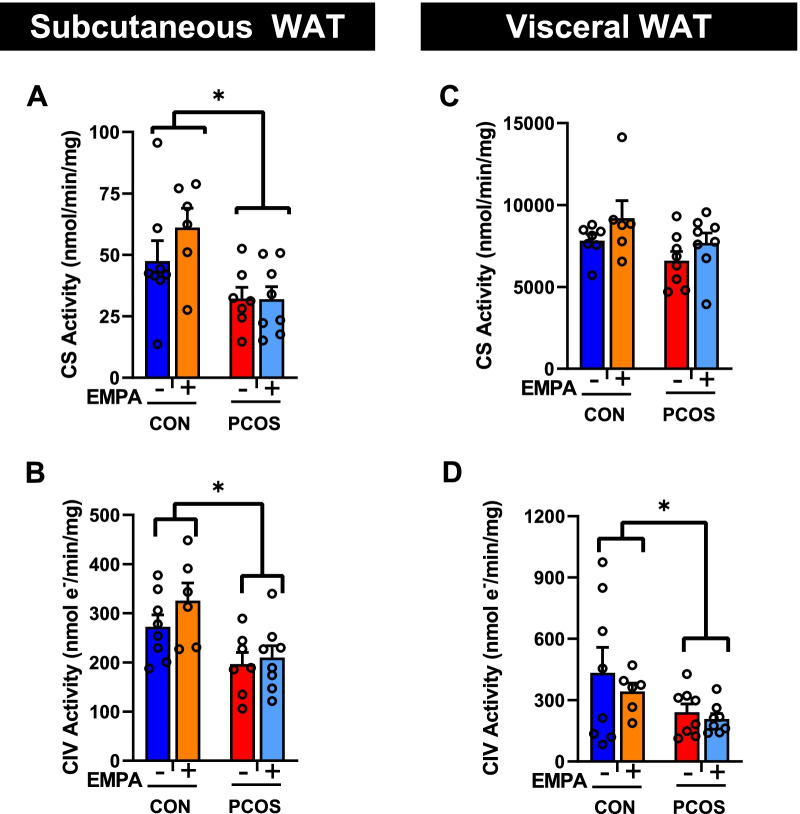
Effect of EMPA on citrate synthase and complex IV activities in white adipose depots in PCOS. Effect of EMPA on subcutaneous WAT **A** citrate synthase (CS) activity and **B** complex IV (CIV) activity and on visceral (retroperitoneal) WAT **C** CS activity and **D** CIV activity after 3 weeks of EMPA treatment. Data normalized by protein content. Data are expressed as mean ± SEM and were analyzed by two-way ANOVA followed by Tukey post hoc tests. No significant interaction was observed by two-way ANOVA. *n* = 6–8 per group

As shown in Fig. 7A, B, in SAT, PCOS overall had lower citrate synthase (*P* < 0.01) and complex IV (*P* < 0.01) activities compared to controls. Compared to EMPA + CON rats, EMPA + PCOS rats had lower citrate synthase activity (32 ± 5 vs 61 ± 8 nmol/min/mg protein, *P* < 0.05) and Complex IV activity (210 ± 24 vs 326 ± 36 nmol e^−^/min/mg protein, *P* < 0.05). In rWAT, PCOS overall had lower complex IV activity (*P* < 0.05), but PCOS had no significant impact on citrate synthase activity (Fig. 7C, D). EMPA had no impact on these two activities in VAT (rWAT) in either group.

### Fatty acid oxidation enzyme gene expression in PCOS model: effect of EMPA

**Fig. 8:**
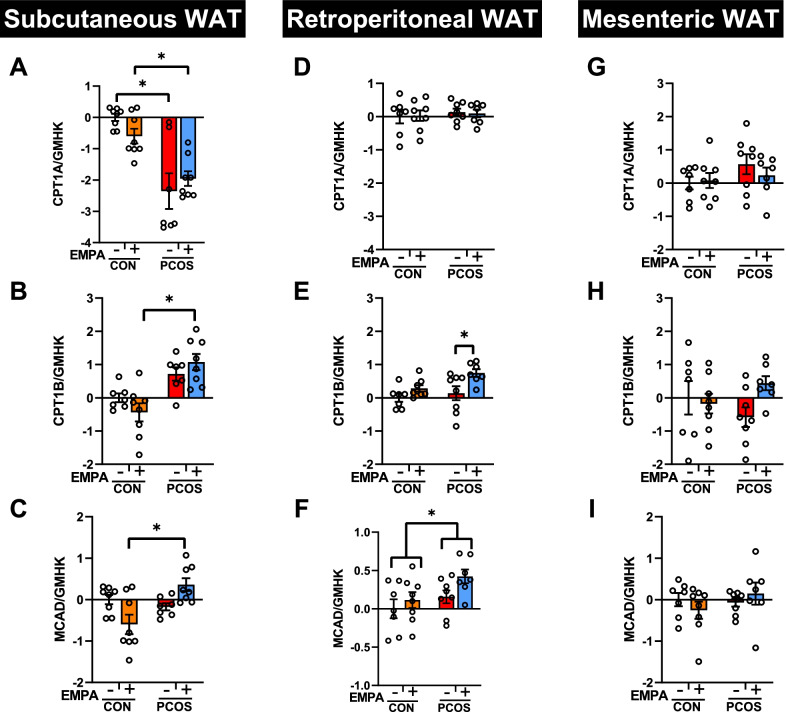
Effect of EMPA on mRNA expression on major components of fatty acid oxidation in white adipose depots in PCOS. Effect of EMPA on subcutaneous white adipose tissue (WAT) mRNA expression of **A** carnitine palmitoyltransferase 1A (CPT1A), **B** carnitine palmitoyltransferase 1B (CPT1B), and **C** medium-chain acyl-CoA dehydrogenase (MCAD); retroperitoneal WAT mRNA expression of **D** CPT1A, **E** CPT1B, and **F** MCAD; and mesenteric WAT mRNA expression of **G** CPT1A, **H** CPT1B, and **I** MCAD after 3 weeks of EMPA treatment. Expression was normalized by the geometric mean of three housekeeping genes (GMHK) and standardized to untreated control rats. Log2 values are expressed as mean ± SEM. Data were analyzed by two-way ANOVA followed by Tukey post hoc tests. Significant interaction was only observed for MCAD in subcutaneous WAT. *n* = 7–8 per group

As seen in Fig. 8A–C, in SAT, PCOS rats had decreased CPT1A mRNA expression compared to controls (− 2.36 ± 0.57 vs 0.00 ± 0.11, *P* < 0.0001) with no significant change in CPT1B or MCAD mRNA expression. EMPA treatment did not modify CPT1A and CPT1B mRNA expression in SAT. PCOS + EMPA rats had a higher expression of MCAD mRNA (0.36 ± 0.16 vs − 0.60 ± 0.24, *P* < 0.01) compared to CON + EMPA rats. Furthermore, in the visceral WAT (retroperitoneal and mesenteric) depots, there were no significant changes in CPT1A by DHT or EMPA treatment (Fig. 8D and G). However, in the rWAT, EMPA significantly increased CPT1B expression in PCOS (0.75 ± 0.11 vs 0.14 ± 0.21, *P* < 0.05) with no effect in controls (Fig. 8E). A similar trend was observed with CPT1B mRNA expression in mWAT between PCOS + EMPA vs PCOS (0.44 ± 0.21 vs − 0.58 ± 0.30, *P* = 0.17), though this was not significant (Fig. 8H). DHT overall increased MCAD mRNA expression in rWAT (*P* < 0.05) with no significant effect of EMPA overall (Fig. 8F). In mWAT, neither DHT nor EMPA treatment changed MCAD mRNA (Fig. 8I).

## Discussion

Women with PCOS have an increased prevalence of obesity-associated cardiometabolic risk factors. Weight loss by lifestyle modifications or bariatric surgery improves cardiometabolic complications in PCOS. However, weight loss is difficult to achieve and sustain, thereby new therapeutic agents are needed. The mechanisms by which androgens expand white adipose tissue, as well as the effect on cardiometabolic risk factors of therapeutic agents used in the clinic, remain unclear. The present study demonstrates that expansion of WAT in PCOS rats is due to an increase in the relative frequency in small-size adipocytes in SAT while there is a decrease in the relative frequency in small-size adipocytes in VAT, demonstrating a differential effect of hyperandrogenemia on adipocytes based on fat depot. We also demonstrate that hyperandrogenemic female rats have mitochondrial dysfunction in WAT. Furthermore, with SGLT2 inhibition, there is an increase in the frequency of small adipocytes in both VAT depots but not in SAT in PCOS rats. Meanwhile, in controls, SGLT2 inhibition increases the frequency of small adipocytes in both SAT and VAT depots, though this is not statistically significant in rWAT. In PCOS, mitochondrial SOD mRNA is decreased in both SAT and VAT and SGLT2 inhibition increases mitochondrial SOD mRNA and protein expression in SAT but not in VAT. However, oxidative stress is not decreased in either SAT or VAT in both EMPA-treated groups. Thus, while mitochondrial dysfunction is present in both SAT and VAT of PCOS rats, SGLT2 inhibition does not appear to decrease adiposity by improving mitochondrial function or oxidative stress in WAT in PCOS rats.

In a worldwide survey of women with PCOS, being overweight and difficulty losing weight was the top concern reported [47]. Expansion of the adipose tissue can occur via an increase in adipocyte number (hyperplasia) or size (hypertrophy) [40]. Women are known to preferentially increase SAT instead of VAT until after menopause while men are much more likely to expand their VAT at younger ages [48]. The data of the fat distribution in women with PCOS are conflicting where the VAT may be unchanged [49], increased [50], or decreased [51]. It is well established that the hyperandrogenemic PCOS model has increased fat mass [14, 52, 53]. Our study confirms previous observations in the WAT of the visceral compartments in the PCOS model [53]. Moreover, our study shows an increase in frequency in small adipocytes and a decrease in medium adipocytes in SAT in the PCOS model. This is similar to previous findings that women with PCOS and normal weight had decreased subcutaneous adipocyte size compared to BMI-matched controls [54]. While it is well-known that large adipocytes are associated with insulin resistance and inflammation, the role of small-size adipocytes is unknown. A recent review by Stenkula and Erlanson-Albertsan [55] demonstrated that very small subcutaneous adipocytes in humans have also been associated with IR, which may be because those adipocytes do not store triglycerides. In that case, triglycerides would have to be stored in other WAT depots or visceral organs [17].

Why androgen excess has a different response on adipocytes in SAT versus VAT is unclear, though PPARγ may explain it in part. In our study, we find that PPARγ mRNA is downregulated in mWAT while it is unchanged in sWAT between PCOS and control rats. A downregulation of PPARγ would be in line with decreased hyperplasia, so the mWAT would have to use hypertrophy to expand. However, in the SAT, our data do not show a difference in PPARγ mRNA expression between PCOS and controls. Our findings are in line with recent findings in the sheep model of PCOS where androgens exhibit differential depot-specific adipose dysfunction [56]. Thus, the androgen-mediated downregulation of PPARγ in VAT could be one of the mechanisms by which androgens promote hypertrophy of VAT and IR in PCOS [4, 14]. The molecular mechanisms by which androgens promote adipogenesis in SAT remain unclear.

We have previously published that EMPA treatment decreases fat mass and plasma leptin in PCOS rats without modifying classical cardiovascular risk factors such as IR and lipid levels [14]. In PCOS, there is resistance to the anorexigenic effect of leptin [8]. In this study, we report that EMPA treatment only increases frequency of small adipocytes in the VAT of PCOS, which could explain why EMPA-treated rats have a lower level of leptin compared to untreated PCOS rats [14]. Moreover, we find that PCOS + EMPA rats eat significantly more than untreated PCOS rats, consistent with previous findings in a male rat model of diet-induced obesity [57]. This increase in food intake could be a compensatory adaptation due to the glucosuric effect of EMPA. Of note, fasting plasma glucose, insulin sensitivity, and hemoglobin A1c are also unchanged by EMPA in both treated groups [14]. Moreover, in our present study, the net glucose intake was similar in both treated EMPA groups, suggesting a different mechanism than EMPA-mediated metabolic adaptation. Interestingly, it has been shown that if food intake is restricted, an additional weight loss of 9% can be achieved with EMPA treatment in an experimental model of obesity [57]. The mechanisms by which SGLT2 inhibition increases food intake in the presence of obesity and/or hyperandrogenemia remain unclear at the present.

Women with PCOS have decreased mitochondrial DNA, a marker of mitochondrial content, in circulating leukocytes [21, 22]. To investigate the adipose tissue mitochondrial function in the PCOS model, we explored mitochondrial content as well as mitochondrial processes such as fatty acid oxidation and management of oxidative stress. Gene expression of mitochondrial biogenesis markers, such as PPARγ, PGC1α, and NRF1, were examined in SAT and VAT. In SAT, only NRF1 expression is lower in the PCOS model, and both NRF1 and PPARγ mRNA expressions are elevated by EMPA treatment in PCOS. These gene expression data would suggest that positive regulators of mitochondrial content are decreased in PCOS in both SAT and VAT. Our data with citrate synthase activity, a marker of mitochondrial content [43], suggest that there is decreased mitochondrial content in SAT in PCOS, though no improvement is observed with EMPA. This is further observed with complex IV activity, a marker of mitochondrial oxidative phosphorylation capacity [43]. Complex IV activity is decreased in SAT with PCOS with no improvement with EMPA, even though NRF1 is known for upregulating the expression of complex IV subunits [36]. This could be due to differences between gene and protein expression. Furthermore, EMPA was only given for 3 weeks while DHT was given for 3 months, so there could be a change in protein expression and activity if EMPA were given for a longer period. Meanwhile**,** in VAT, there is no significant difference in citrate synthase activity in PCOS, though complex IV activity is lowered in PCOS. This highlights another difference in how female androgen excess differentially affects VAT compared to SAT. Also, in accordance with the gene expression data, EMPA has no effect on mitochondrial content or oxidative phosphorylation capacity in VAT.

Women with PCOS have increased circulating markers of oxidative stress compared to BMI-matched controls [27], and there is increased ROS in the oocytes of a mouse model of PCOS [23]. To explore oxidative stress in WAT in PCOS, we measured TBARS and antioxidant enzyme expression. In the PCOS model in SAT, we find that mitochondrial and cytosolic SOD are downregulated. This is accompanied by an increase in catalase, which could be a compensatory mechanism. Upregulation of catalase may explain why PCOS rats have a comparable SAT TBARS compared to controls. EMPA in PCOS in SAT increases expression of both cytosolic and mitochondrial SOD, and they have comparable catalase protein expression to untreated PCOS rats. EMPA in PCOS does not affect SAT TBARS, which suggests that this increase in SOD does not significantly change oxidative stress in SAT any more than the increased catalase already present in PCOS does.

We also measured urinary isoprostane excretion and plasma total antioxidant capacity as systemic markers of oxidative stress. We find that at baseline at 12 weeks of age that PCOS rats actually had decreased urinary isoprostane excretion compared to controls, though this difference disappeared by 16 weeks of age. However, both control and PCOS rats treated with EMPA have increased urinary isoprostanes compared with their untreated counterparts. As the untreated rats decreased their isoprostane excretion between 12 and 16 weeks of age, we do not know if EMPA actively increased urinary isoprostane excretion or if it simply clamped them. Future experiments are needed to confirm this finding using gas chromatography–mass spectroscopy [58] and to uncover this mechanism. We also find that EMPA does not affect plasma total antioxidant capacity in PCOS. The data with urinary excretion of isoprostanes agree with the WAT TBARS and plasma total antioxidant capacity in that EMPA treatment did not decrease markers of oxidative stress in this PCOS model. SGLT2 inhibitors have been previously reported to decrease oxidative stress in male rodents modeling T2DM in the heart, blood, and urine [38, 59–61]. Regarding fatty acid oxidation in WAT, in male mice with T2DM, EMPA upregulated CPT1B and MCAD [34]. In the present study in SAT, rWAT, and mWAT, EMPA does not affect CPT1A in PCOS, but it significantly increases CPT1B in rWAT with similar trends in mWAT. We also find no significant effect of EMPA in PCOS on MCAD expression. Thus, our findings suggest that biological sex is an important variable in the effect of SGLT2 inhibitors on oxidative stress and fatty acid oxidation.

Whether or not mitochondrial dysfunction first causes fat mass expansion or whether an expansion of fat mass first causes mitochondrial dysfunction is uncertain. In our study, we show that hyperandrogenemic female rats have mitochondrial dysfunction in white adipose tissue. SGLT2 inhibition does not modify mitochondrial content in SAT and VAT. In PCOS, mitochondrial SOD gene expression is downregulated in both SAT and VAT, though this is not mirrored in markers of oxidative stress in this study. Furthermore, SGLT2 inhibition upregulates mitochondrial SOD expression in SAT but not in VAT; however, SGLT2 inhibition does not decrease oxidative stress in either SAT or VAT. Thus, while there is mitochondrial dysfunction in both SAT and VAT of hyperandrogenemic female rats, SGLT2 inhibition does not seem to decrease adiposity by improving mitochondrial function in WAT in young hyperandrogenemic female rats.

Our findings are in line with the literature [33] in that SGLT2 mRNA is not expressed in WAT. A possible novel mechanism to further examine EMPA’s action on WAT is via inhibition of glucose transporter-1 (GLUT1) and glucose transporter-4 (GLUT4). A recent study by Li et al. [62] showed with molecular docking that EMPA has a higher affinity for GLUT1 and GLUT4 than for sodium–hydrogen exchanger 1 (NHE1). EMPA and other SGLT2 inhibitors share a glucose moiety [63] that could theoretically bind with GLUT1 or GLUT4. As the action of SGLT2 inhibitors on NHE1 is a popular hypothesis for cardioprotective effects of SGLT2 inhibitors [64–66], data showing that EMPA may preferably bind GLUT1 or GLUT4 over NHE1 are significant in the field. To further validate their findings, Li et al. found that EMPA-incubation decreased glucose uptake in cultured cardiomyocytes even though there is no apparent SGLT2 protein expression in the heart [62]. As GLUT1 and GLUT4 are found on adipocytes [67], EMPA blocking one or both of these transporters may explain the direct action of EMPA on isolated, cultured adipocytes in previous experiments [34].

## Perspectives and significance

Our findings have important clinical relevance. Weight loss is the first-line intervention to ameliorate cardiometabolic complications in PCOS [12]. We recently showed that administration of glucagon-like peptide-1 receptor agonist improved IR in the PCOS model. This effect was accompanied by a reduction of body weight and fat mass, although in these experiments the PCOS rats were post-estrous cycling [52]. In our present study, lowering fat mass by SGLT2 inhibition failed to ameliorate IR [14], mitochondrial dysfunction, and oxidative stress in PCOS, but it increased the relative frequency of small adipocytes in the visceral WAT. The effect of SGLT2 inhibition upon the visceral WAT could be beneficial in women with PCOS, but additional agents should be used to improve their IR. In addition, hyperandrogenemia in PCOS caused expansion of WAT, which was associated with decreases in mitochondrial content and function in SAT and VAT. It may be that targeting mitochondrial function in PCOS will be necessary to improve IR. The presence of hyperandrogenemia in PCOS could modify the therapeutic response to SGLT2 inhibition in women with PCOS.

## Supplementary Information

**Additional file 1: Figure S1.** Representative adipose tissue images. Representative histological images in (A) subcutaneous, (B) retroperitoneal, and (C) mesenteric white adipose tissue. Control and PCOS rats were treated with and without empagliflozin (EMPA). Images were taken at 40× magnification. **Figure S2.** Effect of EMPA on protein expression on antioxidant enzymes in retroperitoneal white adipose tissue in PCOS. Effect of EMPA on retroperitoneal white adipose tissue (WAT) protein expression of (A) cytosolic superoxide dismutase (SOD1, ~ 18 kDa), (B) mitochondrial superoxide dismutase (SOD2, ~ 22 kDa), and (C) catalase (~ 60 kDa) after 3 weeks of EMPA treatment. Data were normalized by total protein content (TPC). Data are expressed as mean ± SEM and were analyzed by two-way ANOVA followed by Tukey post hoc tests. No significant interaction was observed by two-way ANOVA. *n* = 4–5 per group. **Figure S3.** Effect of EMPA on protein expression on antioxidant enzymes in mesenteric white adipose tissue in PCOS. Effect of EMPA on mesenteric white adipose tissue (WAT) protein expression of (A) cytosolic superoxide dismutase (SOD1, ~ 18 kDa), (B) mitochondrial superoxide dismutase (SOD2, ~ 22 kDa), and (C) catalase (~ 60 kDa) after 3 weeks of EMPA treatment. Data were normalized by total protein content (TPC). Data are expressed as mean ± SEM and were analyzed by two-way ANOVA followed by Tukey post hoc tests. No significant interaction was observed by two-way ANOVA. *n* = 4 per group.

## Data Availability

All data generated or analyzed during this study are included in this published article and its additional information files.
