# Supplementary material for: Mitochondrial function and oxidative stress in white adipose tissue in a rat model of PCOS: effect of SGLT2 inhibition
Source: Biol Sex Differ. 2022 Aug 19;13:45. doi: 10.1186/s13293-022-00455-x (PMC9389812; doi:10.1186/s13293-022-00455-x)
Supplement: Supplementary file 1 — Additional file 1: Figure S1. Representative adipose tissue images. Representative histological images in (A) subcutaneous, (B) retroperitoneal, and (C) mesenteric white adipose tissue. Control and PCOS rats were treated with and without empagliflozin (EMPA). Images were taken at 40× magnification. Figure S2. Effect of EMPA on protein expression on antioxidant enzymes in retroperitoneal white adipose tissue in PCOS. Effect of EMPA on retroperitoneal white adipose tissue (WAT) protein expression of (A) cytosolic superoxide dismutase (SOD1, ~ 18 kDa), (B) mitochondrial superoxide dismutase (SOD2, ~ 22 kDa), and (C) catalase (~ 60 kDa) after 3 weeks of EMPA treatment. Data were normalized by total protein content (TPC). Data are expressed as mean ± SEM and were analyzed by two-way ANOVA followed by Tukey post hoc tests. No significant interaction was observed by two-way ANOVA. n = 4–5 per group. Figure S3. Effect of EMPA on protein expression on antioxidant enzymes in mesenteric white adipose tissue in PCOS. Effect of EMPA on mesenteric white adipose tissue (WAT) protein expression of (A) cytosolic superoxide dismutase (SOD1, ~ 18 kDa), (B) mitochondrial superoxide dismutase (SOD2, ~ 22 kDa), and (C) catalase (~ 60 kDa) after 3 weeks of EMPA treatment. Data were normalized by total protein content (TPC). Data are expressed as mean ± SEM and were analyzed by two-way ANOVA followed by Tukey post hoc tests. No significant interaction was observed by two-way ANOVA. n = 4 per group. [file 13293_2022_455_MOESM1_ESM.pdf]

Supplemental Figure 1A: Representative Images of Subcutaneous WAT

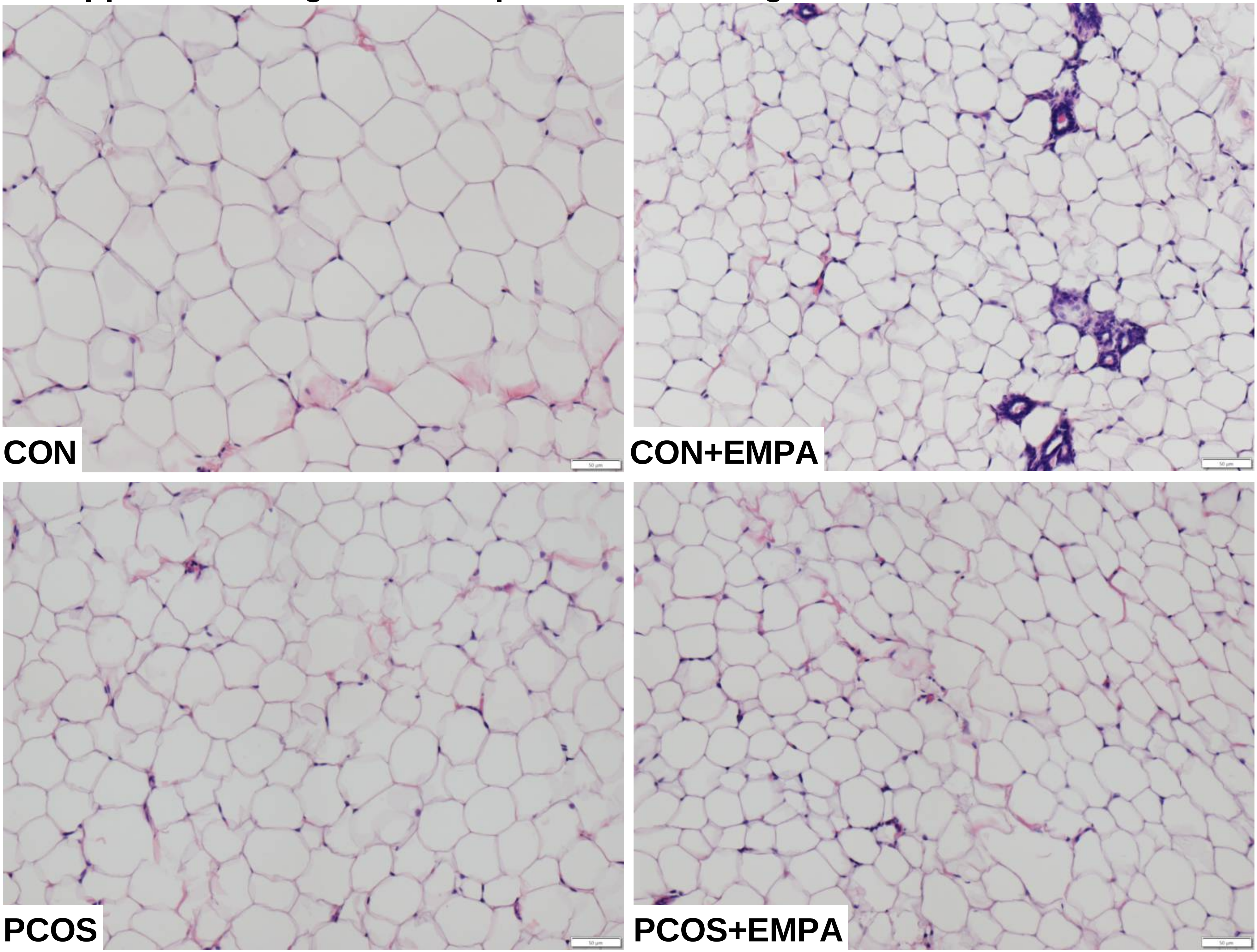

Supplemental Figure 1B: Representative Images of Retroperitoneal WAT

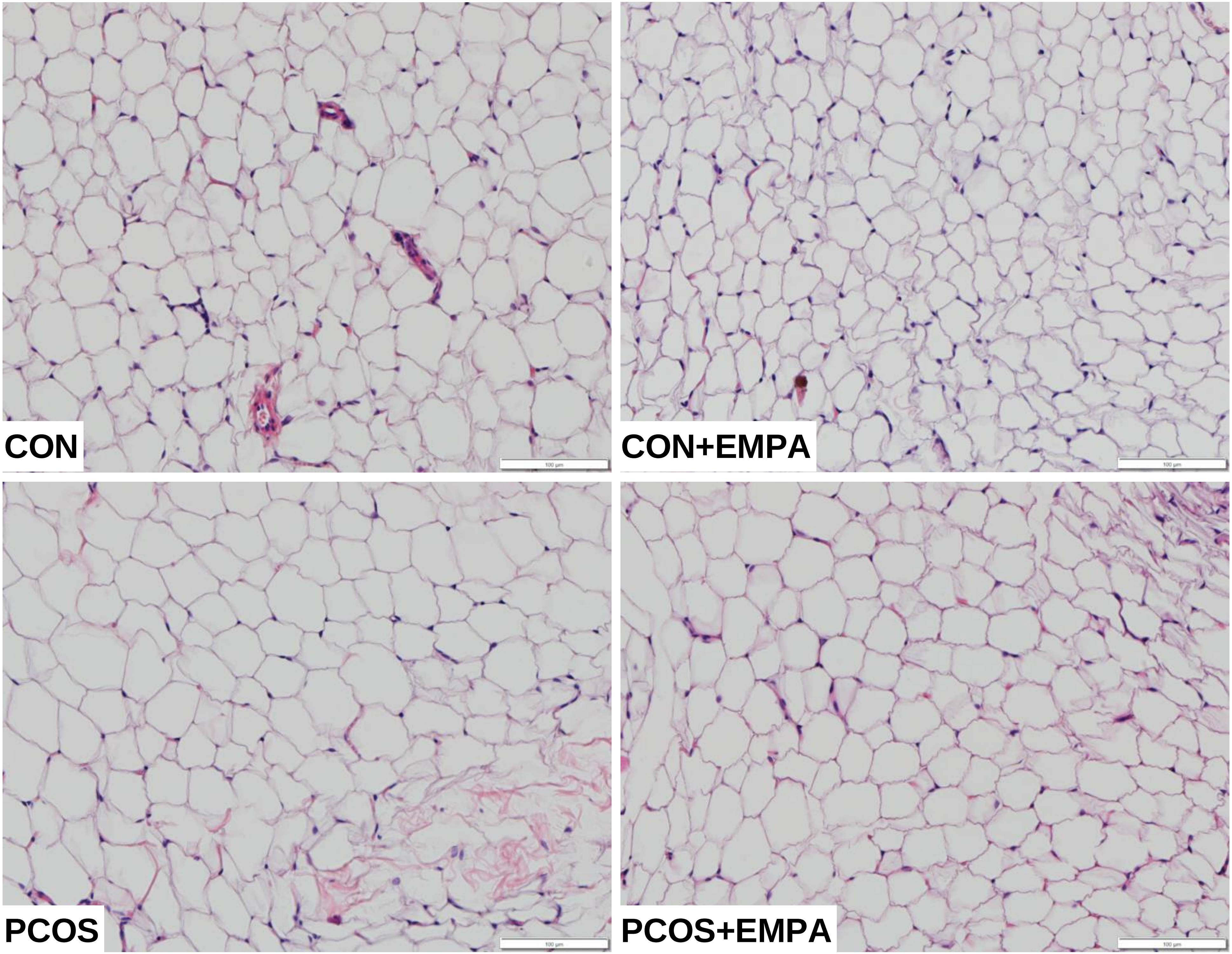

Supplemental Figure 1C: Representative Images of Mesenteric WAT

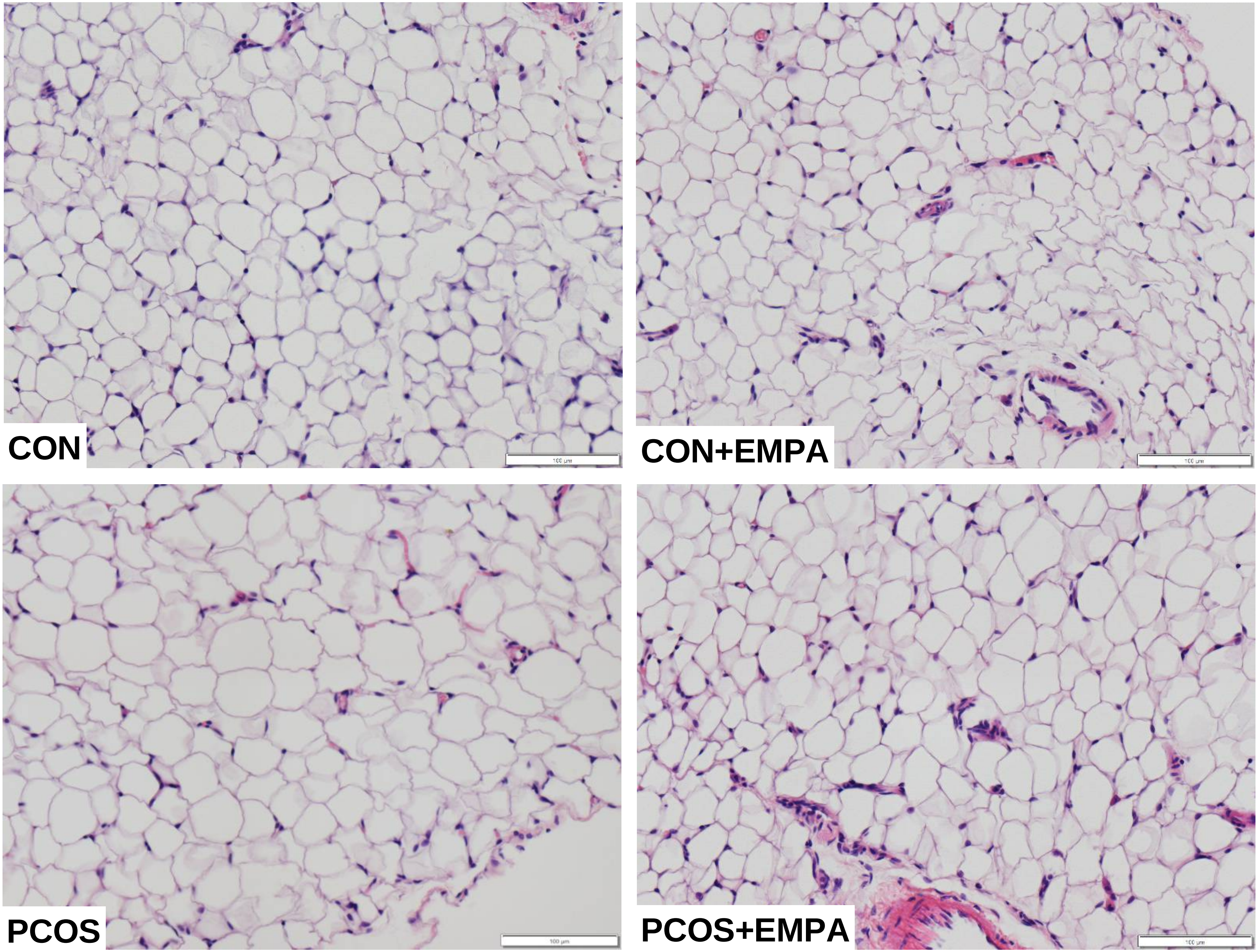

Supplemental Figure 2

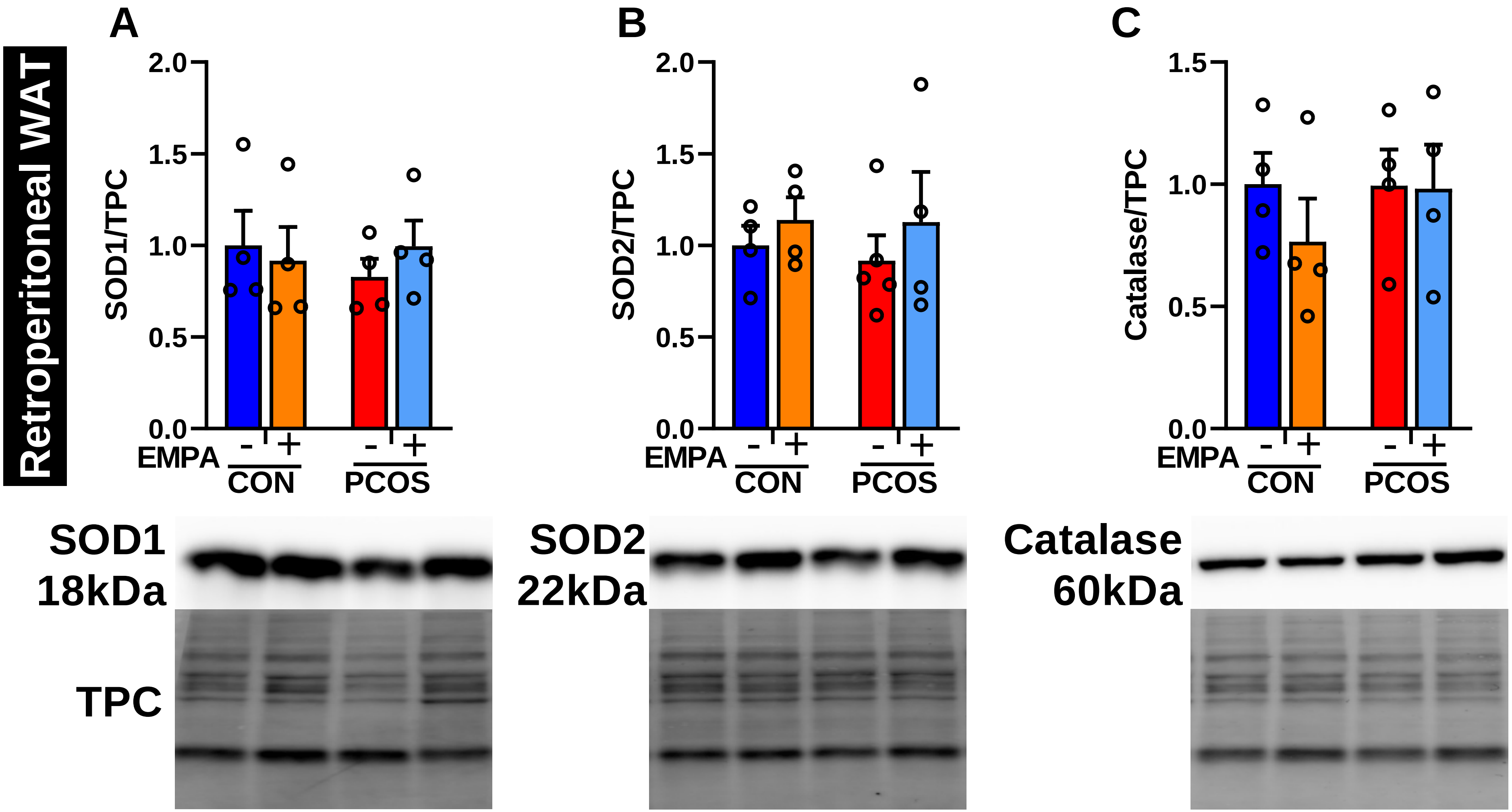

Supplemental Figure 3

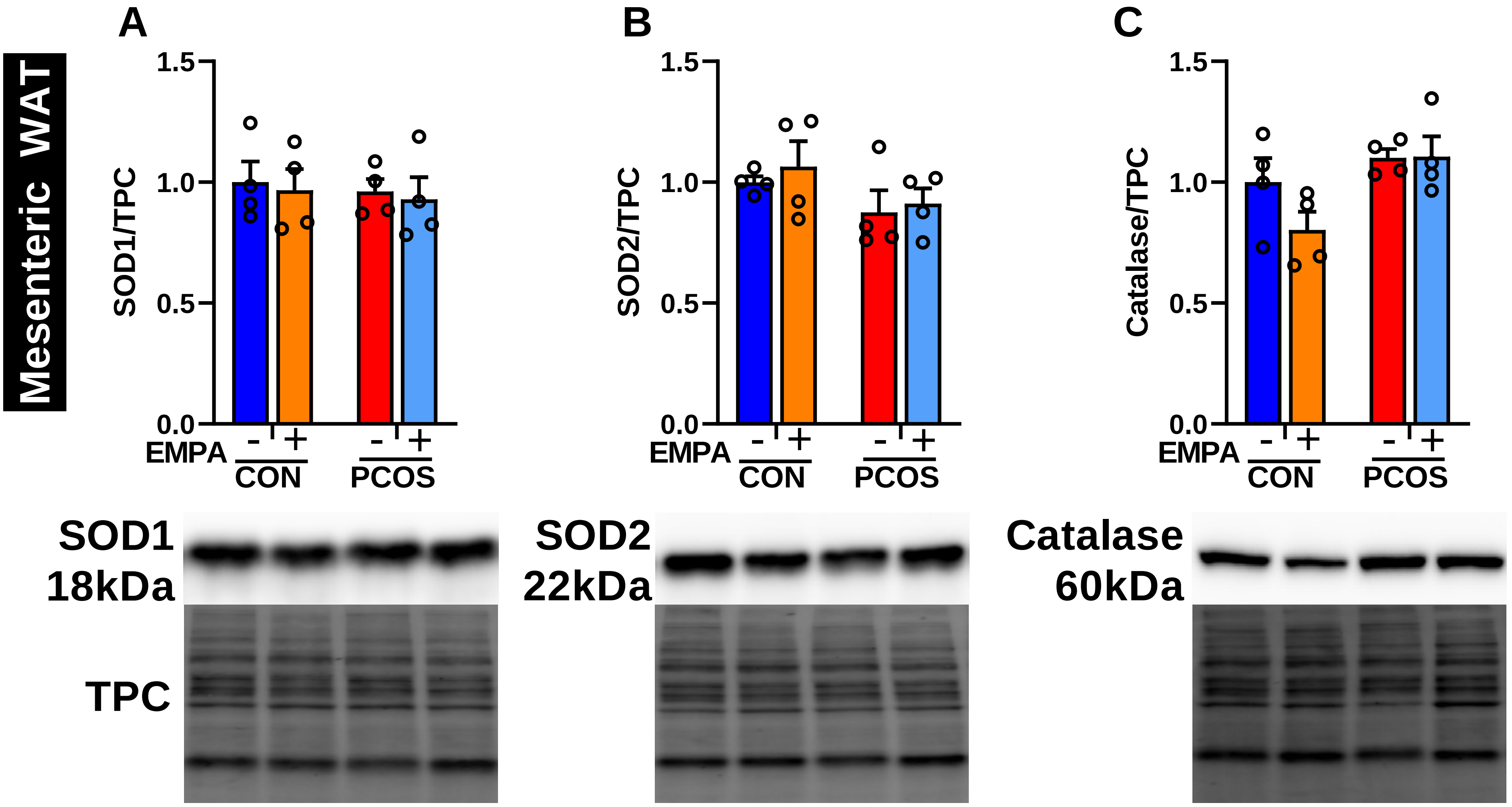

SUPPLEMENTAL FIGURE LEGENDS

Supplemental Figure 1: Representative adipose tissue images. Representative histological images in (A) subcutaneous, (B) retroperitoneal, and (C) mesenteric white adipose tissue. Control and PCOS rats were treated with and without empagliflozin (EMPA). Images were taken at 40x magnification.

Supplemental Figure 2: Effect of EMPA on protein expression on antioxidant enzymes in retroperitoneal white adipose tissue in PCOS. Effect of EMPA on retroperitoneal white adipose tissue (WAT) protein expression of (A) cytosolic superoxide dismutase (SOD1, ~18 kDa), (B) mitochondrial superoxide dismutase (SOD2, ~22 kDa), and (C) catalase (~60 kDa) after 3 weeks of EMPA treatment. Data were normalized by total protein content (TPC). Data are expressed as mean ± SEM and were analyzed by two-way ANOVA followed by Tukey post-hoc tests. No significant interaction was observed by two-way ANOVA. n = 4-5 per group.

Supplemental Figure 3: Effect of EMPA on protein expression on antioxidant enzymes in mesenteric white adipose tissue in PCOS. Effect of EMPA on mesenteric white adipose tissue (WAT) protein expression of (A) cytosolic superoxide dismutase (SOD1, ~18 kDa), (B) mitochondrial superoxide dismutase (SOD2, ~22 kDa), and (C) catalase (~60 kDa) after 3 weeks of EMPA treatment. Data were normalized by total protein content (TPC). Data are expressed as mean ± SEM and were analyzed by two-way ANOVA followed by Tukey post-hoc tests. No significant interaction was observed by two-way ANOVA. n = 4 per group.
